# Novel Radiotherapy Delivery Modalities and Their Potential to Synergize with Immunotherapy

**DOI:** 10.3390/biomedicines14071607

**Published:** 2026-07-17

**Authors:** Ivaylo B. Mihaylov, Benjamin Spieler

**Affiliations:** Department of Radiation Oncology, Leonard M. Miller School of Medicine, University of Miami, Miami, FL 33136, USA; bspieler@miami.edu

## 1. Introduction

The marriage of radiotherapy (RT) and immune checkpoint inhibition (ICI) has been driven, to date, largely by conventional and stereotactic body delivery. Yet a growing array of unconventional radiation strategies—spatially fractionated GRID and LATTICE; very low-dose RT (LDRT); targeted radionuclide therapy (TRT) and theranostics; nanoparticle-mediated radiosensitization; and RT combined with locoregional immunotherapy, including intratumoral ICI and CAR-T—each carry distinct radiobiological signatures that may be uniquely suited to ICI partnering. Spanning the immunogenicity spectrum from “hot” to “cold” tumors, these modalities deserve a more prominent place in the conversation about RT-ICI synergy.

## 2. Spatially Fractionated Radiotherapy Is More than Dose Architecture

Modern external beam radiotherapy (RT) prizes dose uniformity. Spatially fractionated RT (SFRT) deliberately abandons it, sculpting steep intratumoral gradients of high-dose peaks and low-dose valleys (GRID RT). GRID RT, used since the early twentieth century, and its three-dimensional successor LATTICE RT are the prototypical examples, and they have long been justified on the basis that bulky, unresectable tumors—large sarcomas, in particular—cannot be safely dosed homogeneously [1]. What is becoming increasingly clear is that the dose architecture itself may be the more interesting feature.

SFRT engages at least three radiobiological mechanisms: vascular disruption in the high-dose peaks; bystander and abscopal effects driven by soluble mediators such as TNF-α, TRAIL, and ceramide diffusing from peak into valley regions; and direct modulation of the tumor immune microenvironment [2]. Crucially, the peak-to-valley structure of LATTICE simultaneously delivers ablative doses sufficient to trigger immunogenic cell death and neoantigen release, while sub-5 Gy valleys preserve tumor perfusion and the trafficking of newly primed T cells—populations whose depletion is a recognized cost of protracted or multifraction high-dose regimens [3]. In immunocompetent breast tumor models, GRID combined with anti-PD-1 and anti-CTLA-4 has produced antitumor responses extending well beyond the irradiated field, and partial-volume LATTICE has generated more CD3+ infiltration in contralateral tumors than whole-tumor irradiation [3]. A clinical case combining high-dose LATTICE with anti-PD-1 in bulky metastatic non-small-cell lung cancer documented a 78% volume reduction at one month and complete local response at five months in a tumor that had progressed on palliative RT alone [4].

These observations support a model in which SFRT plus immune checkpoint inhibition (ICI) potentiates both local control and systemic immunity. Prospective evaluation will require standardization of peak-to-valley dosimetry across institutions—work that an international consensus framework has begun [1].

## 3. Low-Dose Radiotherapy Turns up the Heat

Ablative RT has dominated the RT-ICI conversation. Very low-dose RT (LDRT; 0.5–2 Gy per fraction) inverts the premise. Immunologically cold tumors—those with sparse T cell infiltration and a stroma dominated by suppressive myeloid cells and regulatory T cells—are largely refractory to ICI and represent a stubborn obstacle in polymetastatic disease.

Herrera and colleagues showed that repeated LDRT at 0.5–2 Gy reprograms cold microenvironments by inducing sustained recruitment of T cells, monocytes, dendritic cells, and NK cells in an interferon-dependent manner [5]. In preclinical ovarian models, weekly total abdominal LDRT at 1 Gy produced maximal immune infiltration and the highest CD8/Treg ratios. A phase I trial combining LDRT with low-dose cyclophosphamide and dual checkpoint blockade yielded objective regressions in 37% of patients with immune-cold gastrointestinal, prostate, and ovarian tumors, with responders showing CD4+ Th1-dominant infiltration and NKG2D-dependent cytotoxicity [5,6].

LDRT therefore offers a means of converting cold tumors into ICI-responsive phenotypes through sub-lethal stress rather than direct killing, and it sidesteps the dosimetric constraints that make ablative coverage impractical in peritoneal carcinomatosis or diffuse bone metastases [5]. More provocatively, the peak-to-valley architecture of GRID and LATTICE may be the only platform that can deliver immunostimulatory low-dose, cGAS/STING-activating intermediate-dose, and ablative high-dose radiation to a single tumor in a single session—engaging the full radiobiological dose–immune spectrum at once.

## 4. Theranostics and Targeted Radionuclide Therapy Take the Conversation Systemic

Targeted radionuclide therapy (TRT) delivers radiation through tumor-selective vectors—small molecules, peptides, or antibodies—and is now approved in neuroendocrine tumors (^177^Lu-DOTATATE) and prostate cancer (^177^Lu-PSMA-617). The theranostic paradigm pairs a diagnostic radiolabel with a therapeutic counterpart on the same molecule, enabling dosimetry, patient selection, and response assessment on a single platform [7].

TRT’s most distinctive feature in the metastatic setting is its ability to irradiate every tumor deposit from a single systemic dose—something focal external beam cannot do. Its intratumoral dose distribution is governed by particle path length and linear energy transfer, producing a heterogeneity profile that is fundamentally different from external beam and with potentially different immunological consequences [8]. Alpha emitters such as ^225^Ac and ^211^At deposit dense ionization over short ranges, with reduced oxygen dependence—an attractive property in hypoxic, radioresistant disease [8].

Preclinical evidence supports TRT-ICI synergy. Using ^90^Y-NM600, a pan-tumor alkylphosphocholine analog, tumor-directed TRT at 2.5–5 Gy produced complete responses in 45–66% of mice with ICI-refractory tumors when combined with ICI, compared with zero responses for either agent alone [9]. The mechanism includes STING pathway activation, upregulation of Fas, MHC-I, PD-L1, and DR5, and STING-dependent synergy with anti-CTLA-4 [10]. FAP-targeted TRT, aimed at cancer-associated fibroblasts rather than tumor cells, offers a stromal-reprogramming approach with early evidence of synergy with PD-1/PD-L1 blockade [11]. Immunotheranostics—using PET imaging of immune targets such as PD-L1 with ^68^Ga-WL12 to guide radionuclide therapy—offers a route to in vivo patient stratification for RT-ICI combinations [12]. Clinical evaluation is now underway with ^177^Lu-DOTATATE, ^177^Lu-PSMA-617, and ^223^RaCl_2_ across several diseases [10].

## 5. Nanoparticles Bring Radiosensitization and Drug Delivery into One Platform

Nanoparticles offer something that conventional pharmacology cannot: a single particle that can simultaneously amplify the physical effects of radiation and shuttle an immunotherapeutic payload into the tumor. High atomic number (high-Z) nanoparticles—gold, hafnium oxide (NBTXR3), gadolinium chelates (AGuIX), bismuth—enhance local energy deposition through photoelectric and Auger effects, amplify reactive oxygen species, and disrupt DNA repair [13]. NBTXR3 and AGuIX have reached clinical evaluation, with evidence of amplified tumor cell kill and sparing of adjacent normal tissue [13,14].

Beyond physical dose enhancement, high-Z particles promote immunogenic cell death—calreticulin exposure, HMGB1 release, neoantigen dispersal—amplifying the in situ vaccine effect of RT and providing a mechanistic basis for nanoparticle-RT-ICI triple combinations [15]. A complementary strategy uses nanoparticles as ICI carriers, exploiting the enhanced permeability and retention effect to concentrate anti-PD-1, anti-PD-L1, or anti-CTLA-4 antibodies in the tumor while limiting systemic exposure and immune-related adverse events. Hafnium oxide metal–organic frameworks co-formulated with anti-PD-L1 have elicited systemic antitumor immunity in preclinical models, demonstrating that radiosensitization and checkpoint blockade can be delivered from a single tumor-homing vehicle [15].

## 6. Across the Immunogenicity Spectrum, and Toward Locoregional ICI

Immunotherapy has changed the landscape of clinical cancer management [16]. ICI agents have been utilized alone or in combination with immunotherapy or radiation [17,18]. Their use is still an active area of research, where recently predictive models have been developed outlining the management benefits and drawbacks [19,20,21,22]. There is a lot to be learned, since the function and the interactions of the immune system are very complex. Radiotherapy in particular has a great yet understudied and underutilized potential to positively synergize with ICI and result in more prolonged and durable patient outcomes.

Across the immunogenicity spectrum, from inflamed hot tumors to immune-excluded and immune-desert phenotypes, RT is emerging as a broadly applicable modulator capable of engaging the cancer immunity cycle at multiple nodes [17]. Ablative SBRT amplifies existing antitumor responses through high-dose immunogenic killing. LDRT, at the other extreme, builds the conditions under which T cells can infiltrate. SFRT bridges the two, engaging cGAS/STING in valley zones alongside immunogenic cell death and vascular disruption in peaks—within a single fraction.

Locoregional ICI delivery is a natural complement. Intratumoral or organ-directed infusion achieves high local drug concentrations, primes immunity at the antigen source, and can generate responses beyond the injected lesion while reducing systemic toxicity [23]. A phase I/II trial of low-dose RT plus intratumoral CpG oligodeoxynucleotide in B-cell lymphoma produced both local and systemic anti-lymphoma activity with acceptable tolerability—an early clinical proof of concept for in situ vaccination [24]. Bispecific antibodies engaging tumor antigens and cytotoxic T cells simultaneously may also synergize with RT through MHC-I upregulation and enhanced antigen presentation; anti-TGF-β/PD-L1 bispecifics combined with RT have shown superior microenvironmental remodeling in preclinical models, alongside the attenuation of radiation-induced pulmonary fibrosis [25]. CAR-T cell therapy, hampered in solid tumors by poor trafficking and persistence, may similarly benefit from radiation-driven MHC-I upregulation, neoantigen shedding, and local cytokine release, with preclinical data showing that subtherapeutic local RT improves CAR-T migration and limits antigen-low escape [26].

## 7. The Challenge Ahead

The therapeutic potential of pairing these unconventional radiation modalities with immunotherapy is substantial, but the field’s central difficulty is not the absence of compelling biology—it is the abundance of it. SFRT, LDRT, TRT, theranostics, nanoparticle radiosensitizers, intratumoral ICI, bispecifics, and CAR-T each carry their own mechanistic rationale, and each suits a different clinical situation. The work ahead is rational integration: matching the radiation modality, beam morphology, and dose architecture to the immunological phenotype of the tumor, the pattern of disease distribution, and the patient. That will not be accomplished without prospective, biomarker-enriched clinical trials, conducted in close partnership between radiation oncology, nuclear medicine, immunology, and nanomedicine.

## Data Availability

Available upon request.
